# Differences in Maturation Status and Immune Phenotypes of Circulating Helios^+^ and Helios^−^ Tregs and Their Disrupted Correlations With Monocyte Subsets in Autoantibody-Positive T1D Individuals

**DOI:** 10.3389/fimmu.2021.628504

**Published:** 2021-05-12

**Authors:** Yuyue Zhang, Jie Zhang, Yun Shi, Min Shen, Hui Lv, Shu Chen, Yingjie Feng, Heng Chen, Xinyu Xu, Tao Yang, Kuanfeng Xu

**Affiliations:** Department of Endocrinology and Metabolism, The First Affiliated Hospital of Nanjing Medical University, Nanjing, China

**Keywords:** type 1 diabetes, Tregs, Helios, monocytes, regulatory

## Abstract

CD4 Tregs are involved in the regulation of various autoimmune diseases but believed to be highly heterogeneous. Studies have indicated that Helios controls a distinct subset of functional Tregs. However, the immunological changes in circulating Helios^+^ and Helios^−^ Tregs are not fully explored in type 1 diabetes (T1D). Here, we elucidated the differences in maturation status and immune regulatory phenotypes of Helios^+^ and Helios^−^ Tregs and their correlations with monocyte subsets in T1D individuals. As CD25^−/low^ FOXP3^+^ Tregs also represent a subset of functional Tregs, we defined Tregs as FOXP3^+^CD127^−/low^ and examined circulating Helios^+^ and Helios^−^ Treg subpopulations in 68 autoantibody-positive T1D individuals and 68 age-matched healthy controls. We found that expression of both FOXP3 and CTLA4 diminished in Helios^−^ Tregs, while the proportion of CD25^−/low^ Tregs increased in Helios^+^ Tregs of T1D individuals. Although the frequencies of neither Helios^+^ nor Helios^−^ Tregs were affected by investigated T1D genetic risk loci, Helios^+^ Tregs correlated with age at T1D diagnosis negatively and disease duration positively. Moreover, the negative correlation between central and effector memory proportions of Helios^+^ Tregs in healthy controls was disrupted in T1D individuals. Finally, regulatory non-classical and intermediate monocytes also decreased in T1D individuals, and positive correlations between these regulatory monocytes and Helios^+^/Helios^−^ Treg subsets in healthy controls disappeared in T1D individuals. In conclusion, we demonstrated the alternations in maturation status and immune phenotypes in Helios^+^ and Helios^−^ Treg subsets and revealed the missing association between these Treg subsets and monocyte subsets in T1D individuals, which might point out another option for elucidating T1D mechanisms.

## Introduction

Regulatory T cells (Tregs) are a subset of CD4 T cells that maintain tolerance by exerting the suppression of conventional T cells (1, 2). Forkhead box protein 3 (FOXP3) is the critical transcription factor for the development and suppressive function of Tregs in both human and mouse (3–5). Tregs are also characterized by high expression of the interleukin-2 *α*-chain receptor (IL-2RA, also named as CD25) (6, 7) and low expression of interleukin-7 receptor (IL-7R, also named as CD127) (8). Numerous studies have indicated that the numeric, phenotypic, and functional abnormalities of Tregs are important in the pathogenesis of many autoimmune diseases (9).

Type 1 diabetes (T1D) is an organ-specific autoimmune disease characterized by severe autoimmune destruction of insulin-secreting pancreatic beta cells (10), especially by the combined actions of different immune cells, such as CD4 and CD8 conventional T cells with specificity for islet autoantigens (11). The compromised number and function of Tregs result in the imbalance between Tregs and conventional T cells in T1D individuals, which lead to abnormal immune responses and subsequently T1D development (2, 9). Although multiple lines of evidence suggest a defective function of Tregs and decreased suppression of T effector cells by Tregs in T1D individuals (12–14), the results for the frequency of circulating Tregs were inconsistent in T1D individuals (9). The probable explanation might be that different studies define Tregs with diverse combinations of markers (*e.g.* FOXP3, CD25, and CD127). It is becoming apparent that Tregs are a heterogeneous mixture of cellular phenotypic subtypes that reflect different states of maturation, differentiation, and activation (15–17). Thus, an alteration in frequencies and immune phenotypes of Treg subsets or a shift in the balance between Treg subsets and other immune cells might be present in T1D individuals.

Helios, a member of the Ikaros zinc finger transcription factor family, is selectively expressed in human Tregs, which binds to the FOXP3 promoter, stabilizing FOXP3 expression and increasing Treg suppressive function (18–21). Although Helios is not a biomarker for distinguishing thymic derived Treg (tTreg) and peripherally induced Treg (pTreg) cells (22), studies have indicated that Helios^+^ and Helios^−^ Tregs are two distinct subpopulations in terms of epigenetic changes at the FOXP3 locus, differences in their phenotype and function, and their stability of FOXP3 expression, *etc* (16, 19, 23). However, the immunological changes of either Helios^+^ or Helios^−^ Tregs in T1D individuals are not well clarified in T1D individuals.

In this scenario, we aimed to unravel the differences in frequencies, maturation status, and immune phenotypes of both Helios^+^ and Helios^−^ Tregs in autoantibody-positive T1D compared to age-matched healthy individuals. We also assessed the potential contributing factors affecting these two Treg subpopulations, including T1D genetic risk loci and disease status. Moreover, Helios^+^ and Helios^−^ Treg development may be controlled by differential monocyte subsets with distinct inflammatory cytokines in healthy individuals (24). Therefore, we also investigated the alterations of regulatory monocyte subsets and their correlations with these two Treg subpopulations in T1D individuals.

## Materials and Methods

### Study Participants

This study included the following subjects: 68 unrelated T1D individuals were recruited from the First Affiliated Hospital of Nanjing Medical University. T1D was diagnosed according to the WHO criteria. T1D individuals were enrolled with at least one positive islet-specific autoantibody, including Zinc transporter-8 autoantibody (ZnT8A), glutamic acid decarboxylase autoantibody (GADA), insulinoma-associated-2 autoantibody (IA-2A) or insulin antibody (IAA). ZnT8A, GADA, and IA-2A were measured by radio-binding assays described previously (25), and IAA was measured by ELISA (Biomerica). Sixty-eight age-matched healthy controls were enrolled from the same geographical region without diabetes or overt autoimmune diseases, which were negative for islet-specific autoantibodies. Study size provides sufficient (80%) statistical power to detect a difference between groups at the p = 0.05 level. The clinical characteristics of all the subjects are listed in **Table S1**. All samples were collected with appropriate informed consent from all participants and/or their guardians in a written way. The study was approved by the Ethics Committee from the First Affiliated Hospital of Nanjing Medical University and conducted according to the principles of the Declaration of Helsinki.

### Cell Staining and Multicolor Flow Cytometry

Peripheral mononuclear blood cells (PBMCs) were isolated from whole blood by density gradient centrifugation on Ficoll at study entry and frozen at a core facility. Thawed PBMCs were stained with aqua for live/dead cells, divided equally for the different panels. For Treg panel, thawed cells were stained with surface monoclonal antibodies: CD3 (SK7), CD4 (SK3), CD8 (SK1), CD25 (M-A251), CD127 (A019D5), CD45RA (HI100), CCR7 (GO43H7), and CD28 (CD28.2), then these cells were fixed and permeabilized according to the manufacturer’s instructions (eBioscience) and stained for intracellular FOXP3 (259D/C7), Helios (22F6), and CTLA-4 (BNI3). For monocyte panel, thawed cells were stained with surface monoclonal antibodies: CD14 (HCD14), CD16 (3G8), and HLA-DR (L243). Fluorochrome-conjugated human monoclonal antibodies were purchased from Biolegend or BD Biosciences. Fluorescence Minus One for CD25 was set for Treg panel. Fourteen separate flow cytometry experiments were performed to obtain the data, and one sample from the same healthy individual drawn at the same time was set as a panel control for different experiments. PBMCs were run on FACS Aria II or FACSCalibur (BD Biosciences) and analyzed by FlowJo v10 software (TreeStar, Ashland, OR).

### Genotyping

Genomic DNA was extracted from isolated PBMCs using the DNeasy Blood and Tissue Kit (Qiagen). Genome-wide association studies on T1D have revealed risk loci in/near candidate genes related to Tregs, including rs2104286 in *IL2RA*, rs6897932 in *IL-7R* and rs478582 and rs1893217 in *PTPN2* (from www.t1dbase.org). Here, we assessed these T1D risk loci by TaqMan genotyping assays according to the manufacturer’s protocol. PCR was performed and analyzed on an ABI 7900HT.

### Statistical Analysis

Comparisons between the two groups were evaluated by the Wilcoxon test or Mann–Whitney test for paired or unpaired samples respectively. Correlations were determined using Spearman’s rank test. Statistical analyses were conducted using GraphPad Prism version 7.0, except that multicomponent distributions were performed by partial permutation test using SPICE v5.1. For all comparisons, a *P* value <0.05 was considered significant.

## Results

### A Decreased Expression of FOXP3 in Helios^−^ Tregs, While an Increased Proportion of CD25^−/low^ in Helios^+^ Tregs of T1D Individuals

**Figure 1:**
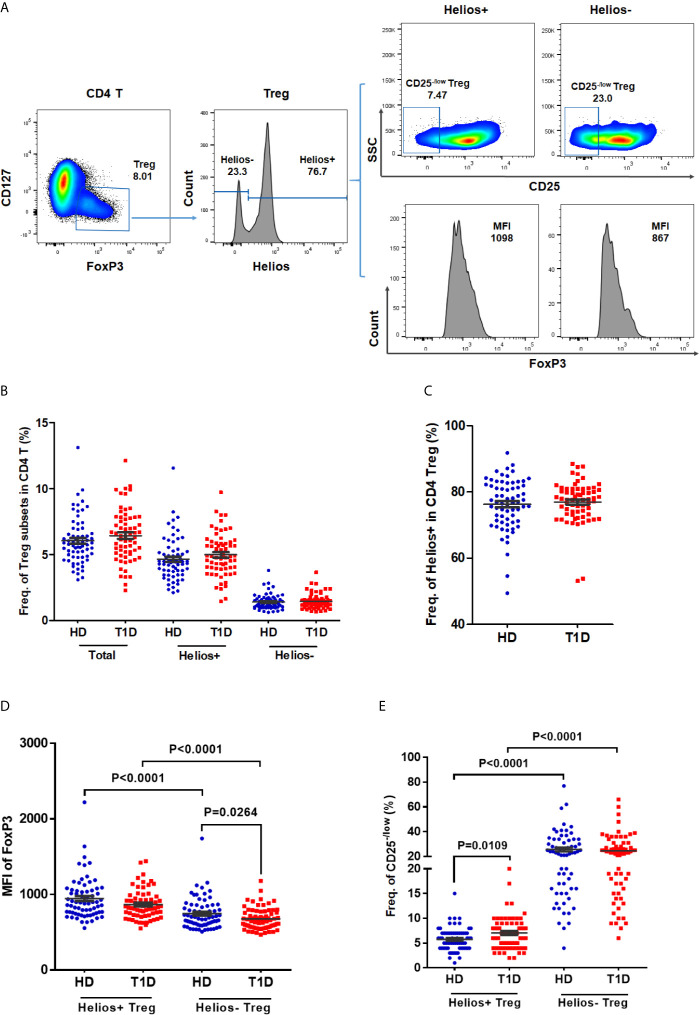
The frequencies of Helios^+^ and Helios^−^ Treg subsets and their expression of FoxP3 and proportions of CD25^− /low^ Tregs in T1D individuals, compared to healthy controls. **(A)** A representative dot plot for gating CD25 and FoxP3 expression in Helios^+^ and Helios^−^ Treg subsets of a healthy donor. **(B)** Evaluation of the percentage of total, Helios^+^ and Helios^−^ Tregs in CD4 T cells between T1D and healthy controls. **(C)** Evaluation of the percentage of Helios^+^ and Helios^−^ Tregs in total Tregs between T1D and healthy controls. **(D)** Differences in mean fluorescence intensity (MFI) of FoxP3 in Helios^+^ and Helios^−^ Tregs of T1D and healthy controls. MFI of FoxP3 was measured to compare the level of expression of this molecule. **(E)** Differences in frequency of CD25^−/low^ in Helios^+^ and Helios^−^ Tregs of T1D and healthy controls. HD, healthy controls. Wilcoxon test was used for statistical comparison between the two different subsets. The results were from 68 autoantibody-positive T1D individuals and 68 age-matched autoantibody-negative healthy controls. Samples from T1D individuals and healthy controls were randomly divided to each independent experiment. One biological sample (from the same healthy donor and drawn at the same time) was performed as control for the experimental reproducibility. Comparisons between T1D and healthy controls were performed by unpaired t test with Welch’s correction. A p value below 0.05 indicates a significant difference between groups.

In humans, CD25^− /low^ FOXP3^+^ Tregs represent a subset of functional Tregs (26, 27), and differential expression of CD127 enables distinction between Tregs (CD127^− /low^) and activated effector T cells (CD127^+^) (8, 28). Therefore, we defined CD4 Tregs as FOXP3^+^CD127^− /low^, and we performed a phenotypic subtype analysis stratified by Helios (16) in T1D individuals. Representative dot plots gating Treg subsets are shown in **Figure 1A**. Compared with age-matched healthy controls, we did not find any difference in the frequencies of total Tregs, Helios^+^, or Helios^−^ Treg subpopulations in autoantibody-positive T1D individuals (**Figures 1B, C**). However, when compared to Helios^+^ Tregs, we observed Helios^−^ Tregs showed much lower expression of FOXP3 (in terms of mean fluorescence intensity, MFI), but much higher proportion of CD25^− /low^ Tregs in both T1D and healthy individuals (**Figures 1D, E**), suggesting reduced stability in Helios^−^ Tregs similar to previous results (16, 21). Interestingly, when compared to healthy individuals, we found a significant decrease in FOXP3^+^ expression (MFI) of Helios^−^ Tregs (*P* = 0.0264) (**Figure 1D**) and an increased frequency of CD25^− /low^ in Helios^+^ Tregs (*P* = 0.0109) (**Figure 1E**) in T1D individuals.

### Frequency of Helios^+^ Tregs in CD4 T Cells Correlated Negatively With Age at T1D Diagnosis and Positively With Disease Duration

**Figure 2:**
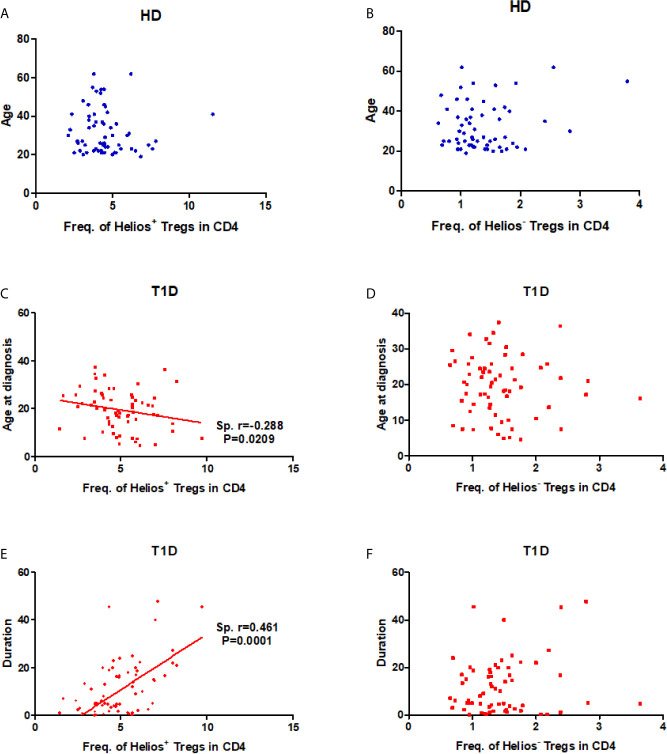
Correlation between frequency of Helios^+^ or Helios^−^ Tregs in CD4 T cells and age at time of blood donation and T1D disease status. **(A, B)** Correlation between frequency of Helios^+^ or Helios^−^ Tregs in CD4 T cells and age at time of blood donation in healthy controls respectively. **(C, D)** Correlation between frequency of Helios^+^ or Helios^−^ Tregs in CD4 T cells and age at T1D diagnosis in T1D individuals respectively. **(E, F)** Correlation between frequency of Helios^+^ or Helios^−^ Tregs in CD4 T cells and T1D duration in T1D individuals respectively. HD, healthy controls. The results were from 68 healthy controls and 68 autoantibody-positive T1D individuals. Samples from T1D individuals and healthy controls were randomly divided to each independent experiment. One biological sample (from the same healthy donor and drawn at the same time) was performed as control for the experimental reproducibility. Spearman correlation was performed for these correlations. A p value below 0.05 indicates a significant difference between groups.

As disease status and genetic risk loci may affect Treg subsets, we investigated the effect of these contributing factors on both Helios^+^ and Helios^−^ Tregs. The results showed no correlation with age at time of blood donation in either Helios^+^ or Helios^−^ Tregs of healthy individuals (**Figures 2A, B**), suggesting they were not affected by age. Interestingly, the percentage of Helios^+^ Tregs in CD4 T cells was positively correlated with T1D disease duration (Spearman *r* = 0.461, *P* = 0.0001) and negatively correlated with age at T1D diagnosis (Spearman *r* = −0.288, *P* = 0.0209) (**Figures 2C, D**). These correlations were not observed in Helios^−^ Tregs of T1D individuals (**Figures 2E, F**). These suggested that the expansion of Helios^+^, but not of Helios^−^ Tregs in CD4 T cells may be related to T1D onset and progression. However, to discern the contributions of four investigated T1D genetic risk loci related to Tregs, we found that none of them affected the frequencies of these two Treg subsets, neither in the absence nor in the presence of T1D disease status (**Figures S1A, B**).

### A Distinct Effector/Memory Differentiation Path Occurs in Helios^+^ Tregs of T1D Individuals

**Figure 3:**
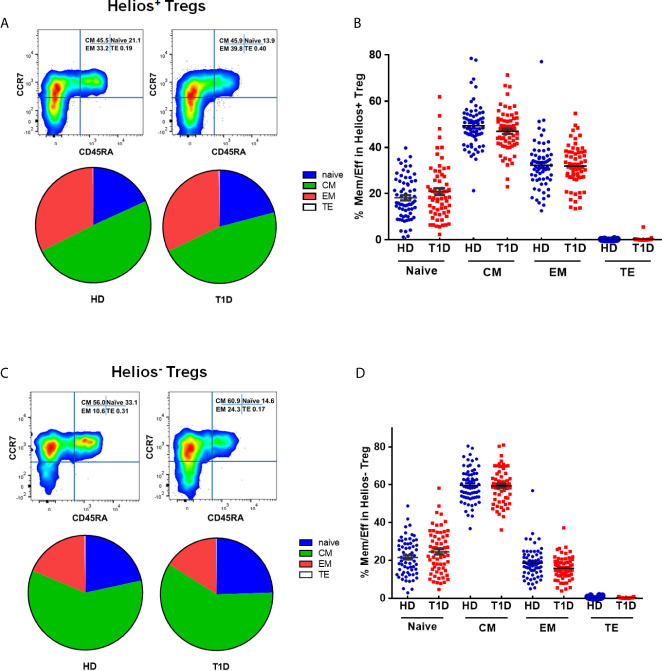
Similar maturation status of both Helios^+^ and Helios^−^ Tregs in T1D individuals, compared with healthy donors (HD). The expression of CD45RA and CCR7 on CD4^+^ T cells from T1D and HD was analyzed by flow cytometry. **(A, C)** Pie charts summarize the data and each slice corresponds to the mean proportion of Helios^+^ and Helios^−^ Tregs for each phenotype. **(B, D)** Possible phenotypes are shown on the x-axis, whereas percentages of distinct T-cell subsets within Helios^+^ and Helios^−^ Tregs are shown on the y-axis. Each point represents a single individual. The results were from 68 autoantibody-positive T1D individuals and 68 age-matched autoantibody-negative healthy controls. Samples from T1D individuals and healthy controls were randomly divided to each independent experiment. One biological sample (from the same healthy donor and drawn at the same time) was performed as control for the experimental reproducibility. Comparisons between phenotype distributions were performed using the partial permutation test, and unpaired t test with Welch’s correction for each phenotype. A p value below 0.05 indicates a significant difference between groups.

The memory/effector characterization might define effector T cell memory pools. Similar to conventional T cells, Treg suppressive capacity may also rely on specific TCR-dependent activation (29). Therefore, Tregs may modify their phenotype, activation, or expansion, which exert different suppressor capacities among distinct Treg differentiation stages. CD45RA does not identify a pure FOXP3^+^Helios^+^ population (30). To explore this issue, we phenotypically discriminated Helios^+^ and Helios^−^ Tregs in terms of CD45RA/CCR7 expression as naïve (N), central memory (CM), effector memory (EM), and terminal effector (TE) Tregs (31–33). As shown in **Figures S2A–D**, compared with Helios^−^ Tregs, Helios^+^ Tregs showed significant differences in memory/effector distributions by a partial permutation test, and increased percentages of EM and diminished proportions of CM subsets irrespective of T1D status (*P* < 0.0001). These data suggested a more differentiated phenotype in Helios^+^ Tregs than in Helios^−^ Tregs. When compared to healthy controls, T1D individuals evinced similar memory/effector distribution by partial permutation test (**Figures 3A, B**) and similar proportions of N, CM, EM and TE subsets (**Figures 3C, D**) in both Helios^+^ and Helios^−^ Tregs, but CM subsets of Helios^+^ Tregs had a tendency to be lower in T1D (P = 0.076).

**Figure 4:**
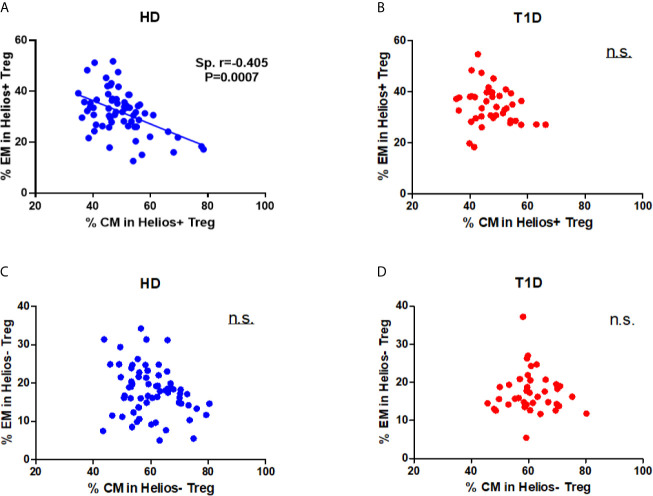
A distinct effector/memory differentiation path occurs in Helios^+^ Tregs from T1D individuals. Correlation analysis between the percentages of CM and EM Treg subsets (Helios^+^ or Helios^−^) from healthy donors (HD) **(A, C)** and T1D **(B, D)** individuals. Spearman rank test was used for the evaluation of the correlation. The results were from 68 healthy controls and 40 autoantibody-positive T1D individuals. Samples from T1D individuals and healthy controls were randomly divided to each independent experiment. One biological sample (from the same healthy donor and drawn at the same time) was performed as control for the experimental reproducibility. Spearman correlation was performed for these correlations. A p value <0.05 was considered as significant. ns, not significant.

Differences in circulating effector/memory subset proportions may occur due to variations in their differentiation path (34). Therefore, we evaluated transitions between effector and memory populations by analyzing correlations between subset proportions. As shown in **Figure 4A**, we observed a strong negative correlation between CM and EM subset proportions for Helios^+^ Tregs in healthy controls (Spearman *r* = 0.405, *P* = 0.0007). However, this correlation was not evident for Helios^+^ Tregs of T1D individuals (**Figure 4B**). In addition, we did not observe any correlation between CM and EM subset proportions for Helios^−^ Tregs in either T1D or healthy individuals (**Figures 4C, D**). These results suggested that transitions of CM and EM Helios^+^ Treg differentiation path are prone to occur in healthy controls, but such a transition in Helios^+^ Tregs disrupted in T1D individuals.

### A Lower Expression of CTLA4 in Helios^−^, but Not Helios^+^ Tregs of T1D Individuals

**Figure 5:**
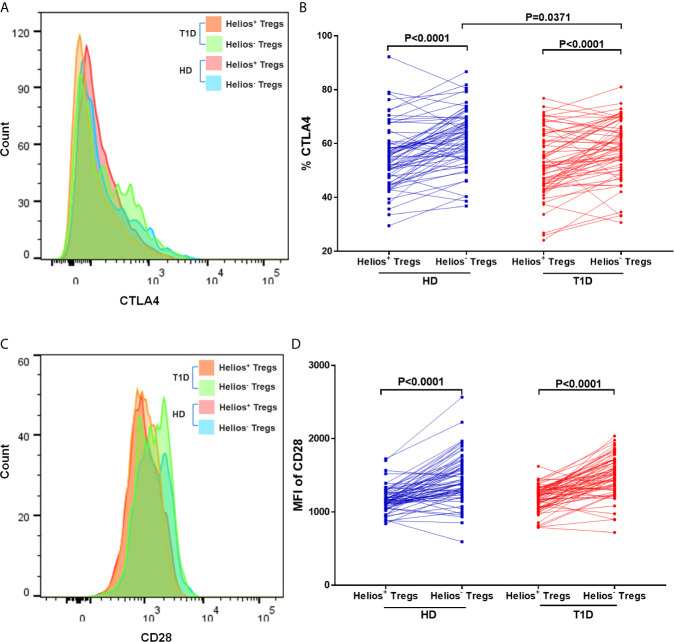
Helios^+^ Tregs differ in CTLA4 and CD28 expression levels with Helios^−^ Tregs. Comparison of CTLA4 **(A, B)** and CD28 **(C, D)** expression between Helios^+^ Tregs and Helios^−^ Tregs from T1D individuals and healthy donors (HD). Wilcoxon rank test was used for paired statistical analysis. The results were from 68 autoantibody-positive T1D individuals and 68 age-matched autoantibody-negative healthy controls. Samples from T1D individuals and healthy controls were randomly divided to each independent experiment. One biological sample (from the same healthy donor and drawn at the same time) was performed as control for the experimental reproducibility. Comparisons between T1D and healthy controls were performed by unpaired t test with Welch’s correction. A p value <0.05 was considered as significant.

We performed a comparative phenotypic analysis for Helios^+^ and Helios^−^ Tregs by evaluating the *ex vivo* expression of CTLA4 and CD28, since they had been associated with regulatory function in Tregs (35). Representative expression of CTLA4 and CD28 in Treg subsets is shown in **Figures 5A, C**. We observed that expression of CTLA4 and CD28 (MFI) was higher in Helios^−^ Tregs compared to Helios^+^ Tregs in both T1D and healthy individuals (*P* < 0.0001) (**Figures 5B, D**), suggesting Helios^+^ and Helios^−^ Tregs are two distinct subpopulations. Continuing our analysis, CTLA4 expression in Helios^−^, but not Helios^+^ Tregs decreased in T1D individuals (*P* = 0.0371) (**Figure 5D**). CD28 expression in Helios^+^ or Helios^−^ Tregs did not differ in T1D individuals compared to healthy controls (**Figure 5C**). These results implicated that Helios^−^ Tregs may display a diminished inhibitory capacity in T1D individuals due to the lower expression of CTLA4.

### Regulatory Monocyte Subsets Balance Are Also Altered in T1D Individuals

**Figure 6:**
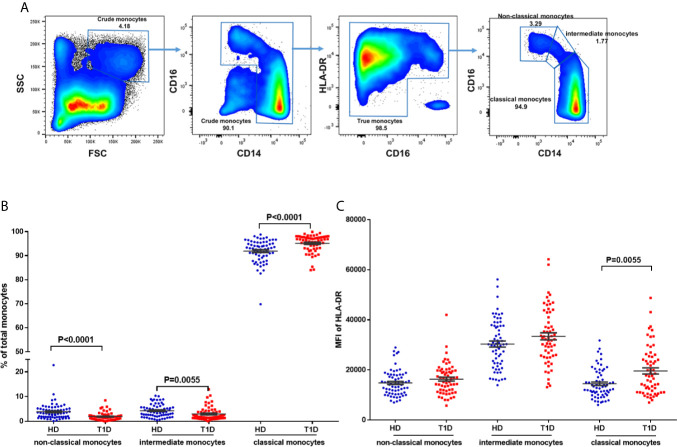
Differences in HLA-DR expression and distribution in circulating monocyte subsets and in T1D compared to age-matched healthy donors (HD). **(A)** Gating strategy for the determination of peripheral monocyte subsets by flow cytometry with the combination of CD14, CD16 and HLA-DR. Three monocyte subsets were defined: non-classical monocytes (CD14^+^CD16^++^), intermediate monocytes (CD14^++^CD16^++^), and classical monocytes (CD14^++^CD16^+^). **(B)** Distribution of monocyte subsets in T1D cases compared to age-matched healthy donors (HD). **(C)** HLA-DR expression in different monocyte subsets of T1D cases compared to age-matched healthy donors (HD). The results were from 68 autoantibody-positive T1D individuals and 68 age-matched autoantibody-negative healthy controls. Samples from T1D individuals and healthy controls were randomly divided to each independent experiment. One biological sample (from the same healthy donor and drawn at the same time) was performed as control for the experimental reproducibility. Comparisons between T1D and healthy controls were performed by unpaired t test with Welch’s correction. A p value <0.05 was considered as significant.

To address this issue, we gated monocytes with the combination of CD14, CD16, and HLA-DR, which could discriminate monocytes from the CD16^+^HLA-DR^−^ NK-cells and neutrophils (36, 37). Representative dot plots for three monocyte subsets are shown in **Figure 6A**, including non-classical (CD14^+^CD16^++^), intermediate (CD14^++^CD16^++^), and classical monocytes (CD14^++^CD16^+^). Our results indicated that age at time of blood donation significantly correlated with the frequencies of these monocyte subsets in both T1D and healthy controls (**Figures S3A–F**); thus it is important to measure the distributions of monocyte subsets in an age-matched manner.

Compared with age-matched healthy controls, we observed decreased proportions of both non-classical and intermediate subsets (*P* < 0.0001 and = 0.0055 respectively) of total circulating monocytes in T1D individuals (**Figure 6B**). We also found that HLA-DR expression (MFI) in intermediate monocytes was significantly higher than in the other two subsets in both T1D and healthy controls, as shown in **Figure 6C**. In addition, HLA-DR expression in classic monocytes, but not intermediate or non-classical monocytes, was significantly increased in T1D individuals (*P* = 0.005). These suggested higher antigen processing and presentation capability of classic monocytes in T1D individuals.

### Correlations Between Regulatory Monocytes and Treg Subsets Disappear in T1D Individuals

**Figure 7:**
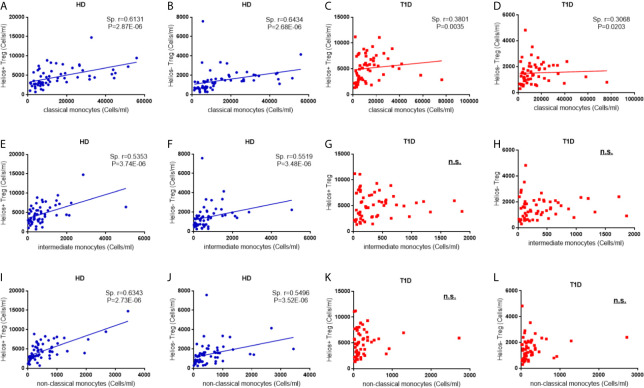
Correlations between regulatory monocytes and Helios^+^ and Helios^−^ Treg subsets in T1D individuals. Correlation analysis between classical, intermediate and classical monocytes and Treg subsets from healthy donors (HD) **(A, B, E, F, I, J)** and T1D individuals **(C, D, G, H, K, L)**. Spearman rank test was used for the evaluation of the correlation. The results were from 68 autoantibody-positive T1D individuals and 68 age-matched autoantibody-negative healthy controls. Samples from T1D individuals and healthy controls were randomly divided to each independent experiment. One biological sample (from the same healthy donor and drawn at the same time) was performed as control for the experimental reproducibility. Spearman correlation was performed for these correlations. A p value <0.05 was considered as significant. ns, not significant.

In the non-infectious setting of chronic autoimmune diseases, cell contact with regulatory T cells is likely to contribute to the regulation of circulating monocytes (24). Therefore, we performed a correlation analysis to investigate the relationship between the absolute numbers of monocyte subsets and Treg subsets. As shown in **Figures 7A, B**, we observe positive correlations between classic monocytes and Treg subsets in both healthy controls (for Helios^+^ subsets, Spearman r = 0.613, *P* = 2.87E-06; for Helios^−^ subsets, Spearman r = 0.643, *P* = 2.68E-06) and T1D individuals (for Helios^+^ subsets, Spearman r = 0.380, *P* = 0.0035; for Helios^−^ subsets, Spearman r = 0.307, *P* = 0.020) (**Figures 7C, D**). Moreover, positive correlations between intermediate monocytes and Treg subsets were observed in healthy controls (for Helios^+^ subsets, Spearman r = 0.535, P = 3.74E-06; for Helios^−^ subsets, Spearman r = 0.552, P = 3.48E-06) (**Figures 7E, F**). Similar correlations were also found between non-classical monocytes and Treg subsets in healthy controls (for Helios^+^ subsets, Spearman r = 0.535, *P* = 3.74E-06; for Helios^−^ subsets, Spearman r = 0.550, *P* = 3.52E-06) (**Figures 7I, J**). However, such correlations between intermediate or non-classic monocytes and Treg subsets were not observed in T1D individuals (**Figures 7G, H, K, L**). Furthermore, the positive correlation between intermediate or non-classic monocytes and total Tregs in healthy controls also disappeared in T1D individuals (data not shown). Interestingly, although these monocyte subsets had no correlation with T1D disease duration, a positive correlation for intermediate monocytes, and a negative correlation for classical monocytes was observed with age at T1D diagnosis respectively (**Figures S3D–F**), which might contribute to the imbalance between regulatory monocytes and Tregs in T1D individuals.

## Discussion

Studies have indicated that Helios^+^ and Helios^−^ Tregs are two distinct functional subpopulations (16, 19, 23), and our results revealed that the frequencies of Helios^+^ and Helios^−^ Tregs were not altered in T1D individuals, which were consistent with those from Du et al. (38). However, other studies indicated that Helios^+^ Tregs were associated with other autoimmune diseases (*e.g.* systemic lupus erythematosus, SLE) (39–41). Furthermore, Zoka et al. did not observe any change in FOXP3 expression in Tregs of T1D individuals (42), but other studies indicated that the stability of FOXP3 in Tregs decreased in T1D individuals (13, 43), and more Tregs from T1D individuals tend to lose Helios expression during the expansion *in vitro* (38). Our results found that decreased expression of FOXP3 only occurred in Helios^−^, but not Helios^+^ Tregs of T1D individuals. Taken together, these results suggested that Helios in Tregs may regulate the development of autoimmune diseases in different orientations, and that FOXP3 expression in Helios^−^ Tregs were more unstable in T1D individuals.

In humans, T1D risk loci are present in key elements of the IL2RA and molecules/phosphatases modulating downstream signaling of IL-2 (*e.g.* PTPN2), which were associated with reduced Treg fitness and/or function in the absence of disease (43–45). Helios regulates IL-2 production in Tregs by silencing IL-2 gene transcription and maintains Treg suppressive function (46). However, we did not find that any of these risk loci affected the frequencies of Helios^+^ or Helios^+^ Tregs in either healthy controls or T1D individuals. As C-peptide levels were influenced by age at T1D diagnosis and disease duration (47), we speculated that an imbalance of Helios^+^ Treg/effector T cells may lead to altered immune attack to islet beta cells, which subsequently influence T1D onset and residual C-peptide levels.

Recent studies have shown that circulating CD25^− /low^ Tregs increased in SLE individuals (26, 48, 49), a finding that was later expanded to T1D (27, 42). Our results revealed that compared to Helios^+^ Tregs, the proportion of CD25^− /low^ Tregs in Helios^−^ Tregs significantly increased in both T1D and healthy controls. We also noted a strikingly significant increase of CD25^− /low^ Tregs in Helios^+^, but not Helios^−^ Treg subsets in T1D individuals. In accordance with our results, increased CD25^− /low^ Treg proportions in CD4^+^FOXP3^+^ or FOXP3^+^Helios^+^ Tregs were also observed in T1D individuals by other authors (42, 50). Moreover, CD25^− /low^ Tregs were derived from CD25^high^ Tregs and were a peripheral marker of recent Treg expansion in response to an autoimmune reaction in tissues (27). Low CD25 expression was also associated with impaired STAT5 phosphorylation upon IL-2 stimulation (50). Taken together, an impaired balance of CD25^high^ and CD25^− /low^ in Helios^+^ Tregs might reflect a decreased late-phase activation of circulating Tregs in T1D individuals.

Similar to conventional T cells, TCR activation induces Tregs differentiation resulting in dissimilar memory populations (51). Mailloux et al. found that most Helios^−^ Tregs were of CM type, while Helios^+^ Tregs are prevalently EM cells, which display a more potent inhibition capacity compared to CM or TE cells (34). However, our results showed that CM cells were the main type for both Helios^+^ and Helios^−^ Tregs irrespective of T1D status. Our results further demonstrated that increases in naive and CM Tregs and a reduction in EM Tregs occurred in both healthy controls and T1D individuals. When compared to healthy controls, we found similarities in memory/effector distribution for Helios^+^ Tregs of T1D individuals, which exhibited a less differentiated phenotype with a decline of high suppressor EM Tregs, probably resulting in increased autoimmune responses. Moreover, a distinct transition between CM and EM Helios^+^ Treg differentiation path occurred in T1D individuals. These differences may lead to dysregulation of Tregs in T1D individuals, which deserves further confirmation in other autoimmune diseases.

Another important issue is the phenotypic differences in Treg subsets. CTLA-4, a critical immunosuppressive regulator of T cell responses, is constitutively expressed on Tregs, and its intracellular domain is very important for Treg biology (35). Dysregulated expression of CTLA-4 leads to immune homeostasis imbalance and autoimmune diseases (52). However, expression of CTLA-4 in Tregs was inconsistent in T1D individuals. Zóka et al. found that CTLA-4 expression in Tregs did not alter (42), while Lindley et al. indicated a significant increase of intracellular CTLA-4 in Tregs of T1D individuals (53). Our results demonstrated a significant decrease of intracellular CTLA-4 in Helios^−^ Tregs, but not in Helios^+^ Tregs of T1D individuals. Combined with the decreased expression of FOXP3, Helios^−^ Tregs tended to be in an unstable status and were easier to lose their immunosuppressive function in T1D individuals.

Although Himmel et al. did not find any significant difference in CTLA-4 expression between Helios^+^ and Helios^−^ nTregs (54), our results revealed that compared to Helios^+^ Tregs, expression of CTLA-4 in Helios^−^ Tregs was strikingly higher in both T1D and healthy individuals. In addition, CD28 is also vital for the activation, homeostasis, or survival of Tregs (55). Similar to the results of CTLA-4 expression, we also found strikingly higher expression of CD28 in Helios^−^ Tregs in both T1D and healthy individuals. As CD28 is the primary driver of Treg proliferation and CTLA-4 functions as the main brake (56), it implied that although the function of Helios^−^ Tregs was unstable and decreased in autoimmune disease status, they might have more immunosuppressive function compared to Helios^+^ Tregs.

Skewed circulating monocytes are also recognized as a heterogeneous population with potentially diverse immune regulatory properties (36, 37). Alternations in monocyte subsets have been described in several autoimmune diseases, including T1D, but different studies yielded contrary results. Irvine et al. reported that the proportions of intermediate monocytes were decreased, while non-classical monocytes increased in total monocytes of both recent-onset and long-standing T1D individuals (57). In comparison, Ren et al. found that the frequencies of classical monocytes decreased, and those of both non-classical and intermediate monocytes increased in T1D individuals (58). However, contrary to their results, we found a significant increase in the proportions of both non-classical and intermediate monocytes in T1D individuals. The explanations for this difference might be as follows. Firstly, unlike previous studies only used the combination of CD14 and CD16, we identified monocyte subsets expressing HLA-DR, which could exclude neutrophils and NK-cells gated with CD14^+^HLA-DR^low/neg^ (37). Secondly, patient risk factors may affect monocyte subsets. For instance, changes in non-classical monocytes were dependent on the age of the patients (59). Our results further revealed that all monocyte subsets are affected by age at time of blood donation in healthy individuals. Thus, it is vital to assess monocyte subsets with age-matched healthy controls. In addition, different from Ren et al. (58), we also found that classical, but not the other two regulatory monocyte subsets in T1D individuals expressed higher levels of HLA-DR, which suggested better antigen presentation capability and led to more autoimmune responses in T1D disease status. The frequencies, immune phenotypes, and function of these monocyte subsets deserve further confirmation by other studies.

Recent studies also followed with interest in the relevance between Tregs and monocyte subsets. Zhang et al. found that the development of Helios^+^ and Helios^−^ Tregs was controlled by CD16^+^ and CD16^-^ monocytes respectively (58). RA subjects had altered Helios^+^ Treg numbers (60), which may be explained by changes in their monocyte subsets (61). In HIV-infected individuals, the frequency of intermediate monocytes was inversely correlated with the frequency of CD45RA^+^ Tregs, especially FOXP3^+^Helios^+^CD45RA^+^ Tregs (62, 63). Different from previous studies, our results demonstrated that the absolute number of the three monocyte subsets had a positive correlation with both Helios^+^ and Helios^−^ Tregs in healthy controls, but such relevance was disrupted in T1D individuals, especially that the correlations disappeared in intermediate and non-classical monocyte subsets. We speculated that such disruption might contribute to T1D pathogenesis, and it points out another option for elucidating T1D mechanisms.

Our study has some limitations. Several studies in mouse models have indicated that the number/percentage/phenotype of pancreas-resident Treg subsets is different from the spleen (64, 65), potentially suggesting that the findings in circulating PBMCs may not represent what happens in the pancreas. This limitation is intrinsic to human studies for ethical reasons. Another limitation is that the study evaluates the expression of two markers only (CTLA-4 and CD28) in the considered Treg subsets.

In conclusion, combined with the decreased expression of FOXP3, Helios^−^ Tregs tend to be unstable and might be easier to lose their immunosuppressive function in T1D individuals. But they might have more immunosuppressive function compared to Helios^+^ Tregs. And Helios^+^ Tregs tended to have a distinct effector/memory differentiation path in T1D individuals. Furthermore, we speculate that the missing association of Treg subsets and monocytes might contribute to T1D pathogenesis, and it points out another option for elucidating T1D mechanisms.

## Data Availability Statement

The original contributions presented in the study are included in the article/**Supplementary Material**. further inquiries can be directed to the corresponding author.

## Ethics Statement

The studies involving human participants were reviewed and approved by the First Affiliated Hospital of Nanjing Medical University. Written informed consent to participate in this study was provided by the participants’ legal guardian/next of kin.

## Author Contributions

KX directed the study design, performed statistical analysis and interpretation of data, and drafted the initial manuscript. YZ and JZ were responsible for the analysis and interpretation of data. MS, HL, SC, and YF contributed to the collection and selection of samples. HC and XX contributed to laboratory measurements. TY gave a critical revision of the manuscript. All authors contributed to the article and approved the submitted version.

## Funding

This study was supported by grants from the National Natural Science Foundation of China (81670715), Jiangsu Province Youth Medical Talents Project (QNRC2016584), the Natural Science Foundation of Jiangsu Province (BK2012486), Jiangsu Government Scholarship for Overseas Studies (JS-2013-260), and the Priority Academic Program Development of Jiangsu Higher Education Institutions (PAPD). 

## Conflict of Interest

The authors declare that the research was conducted in the absence of any commercial or financial relationships that could be construed as a potential conflict of interest.

## Supplementary Material

Supplementary Figure 1 The effect of T1D risk loci on the frequencies of Helios^+^ and Helios^−^ Tregs in both healthy controls and T1D individuals, including rs2104286 in *IL2RA*, rs6897932 in *IL-7R* and rs478582 and rs1893217 in *PTPN2*. Con represents healthy controls. A total of 40 T1D individuals and 40 healthy controls were enrolled for the analysis. For both healthy controls and T1D individuals, comparisons between wild genotype and homozygote+ heterozygote and were performed by unpaired t test with Welch’s correction. A p value below 0.05 indicates a significant difference for a different genotype in each groups.

Supplementary Figure 2 Differing maturation status of Helios^+^ and Helios^−^ Tregs in healthy donors (HD) **(A, B)** and T1D **(C, D)** individuals. Pie charts summarize the data and each slice corresponds to the mean proportion of Helios^+^ and Helios^−^ Tregs for each phenotype. Each point represents a single individual. Possible phenotypes are shown on the x-axis whereas percentages of distinct T-cell subsets within Helios^+^ and Helios^−^ Tregs are shown on the y-axis. The results were from 68 autoantibody-positive T1D individuals and 68 age-matched autoantibody-negative healthy controls. Comparisons between phenotype distributions were performed using the partial permutation test followed by Kruskal–Wallis test and the Dunn’s multiple comparisons post-test, and unpaired t test with Welch’s correction for each phenotype. A p value below 0.05 indicates a significant difference between groups.

Supplementary Figure 3 Correlation between frequency of monocyte subsets and age at time of blood donation and T1D disease status. **(A–C)**, Correlation between frequency of monocyte subsets and age at time of blood donation in healthy controls respectively. **(D–F)**, Correlation between frequency of monocyte subsets and age at T1D diagnosis and T1D duration in T1D individuals respectively. HD, healthy controls. Correlations were determined using the Spearman’s correlation coefficients. The results were from 68 autoantibody-positive T1D individuals and 68 age-matched autoantibody-negative healthy controls. Spearman correlation was performed for these correlations. A p value below 0.05 indicates a significant difference between groups.

## References

[1] Josefowicz SZ Lu LF Rudensky AY. Regulatory T Cells: Mechanisms of Differentiation and Function. Annu Rev Immunol (2012) 30:531–64. 10.1146/annurev.immunol.25.022106.141623 PMC6066374 22224781

[2] Grant CR Liberal R Mieli-Vergani G Vergani D Longhi MS. Regulatory T-cells in Autoimmune Diseases: Challenges, Controversies and–Yet–Unanswered Questions. Autoimmun Rev (2015) 14:105–16. 10.1016/j.autrev.2014.10.012 25449680

[3] Fontenot JD Gavin MA Rudensky AY. Foxp3 Programs the Development and Function of CD4+CD25+ Regulatory T Cells. Nat Immunol (2003) 4:330–6. 10.1038/ni904 12612578

[4] Hori S Nomura T Sakaguchi S. Control of Regulatory T Cell Development by the Transcription Factor Foxp3. Science (2003) 299:1057–61. 10.1126/science.1079490 12522256

[5] Sakaguchi S Miyara M Costantino CM Hafler DA. FOXP3+ Regulatory T Cells in the Human Immune System. Nat Rev Immunol (2010) 10:490–500. 10.1038/nri2785 20559327

[6] Baecher-Allan C Brown JA Freeman GJ Hafler DA. CD4+CD25high Regulatory Cells in Human Peripheral Blood. J Immunol (2001) 167:1245–53. 10.4049/jimmunol.167.3.1245 11466340

[7] Dieckmann D Plottner H Berchtold S Berger T Schuler G. Ex Vivo Isolation and Characterization of CD4(+)CD25(+) T Cells With Regulatory Properties From Human Blood. J Exp Med (2001) 193:1303–10. 10.1084/jem.193.11.1303 PMC2193384 11390437

[8] Liu W Putnam AL Xu-Yu Z Szot GL Lee MR Zhu S. CD127 Expression Inversely Correlates With FoxP3 and Suppressive Function of Human CD4+ T Reg Cells. J Exp Med (2006) 203:1701–11. 10.1084/jem.20060772 PMC2118339 16818678

[9] Qiao YC Pan YH Ling W Tian F Chen YL Zhang XX. The Yin and Yang of Regulatory T Cell and Therapy Progress in Autoimmune Disease. Autoimmun Rev (2017) 16:1058–70. 10.1016/j.autrev.2017.08.001 28778708

[10] Wållberg M Cooke A. Immune Mechanisms in Type 1 Diabetes. Trends Immunol (2013) 34:583–91. 10.1016/j.it.2013.08.005 24054837

[11] Hull CM Peakman M Tree TIM. Regulatory T Cell Dysfunction in Type 1 Diabetes: What’s Broken and How can We Fix it? Diabetologia (2017) 60:1839–50. 10.1007/s00125-017-4377-1 PMC6448885 28770318

[12] Brusko TM Wasserfall CH Clare-Salzler MJ Schatz DA Atkinson MA. Functional Defects and the Influence of Age on the Frequency of CD4+ Cd25+ T-cells in Type 1 Diabetes. Diabetes (2005) 54:1407–14. 10.2337/diabetes.54.5.1407 15855327

[13] Long SA Cerosaletti K Bollyky PL Tatum M Shilling H Zhang S. Defects in IL-2R Signaling Contribute to Diminished Maintenance of FOXP3 Expression in CD4(+)CD25(+) Regulatory T-cells of Type 1 Diabetic Subjects. Diabetes (2010) 59:407–15. 10.2337/db09-0694 PMC2809970 19875613

[14] Long SA Buckner JH. Cd4+Foxp3+ T Regulatory Cells in Human Autoimmunity: More Than a Numbers Game. J Immunol (2011) 187:2061–66. 10.4049/jimmunol.1003224 PMC3160735 21856944

[15] Mason GM Lowe K Melchiotti R Ellis R de Rinaldis E Peakman M. Phenotypic Complexity of the Human Regulatory T Cell Compartment Revealed by Mass Cytometry. J Immunol (2015) 195:2030–7. 10.4049/jimmunol.1500703 26223658

[16] Sebastian M Lopez-Ocasio M Metidji A Rieder SA Shevach EM Thornton AM. Helios Controls a Limited Subset of Regulatory T Cell Functions. J Immunol (2016) 196:144–55. 10.4049/jimmunol.1501704 PMC4685018 26582951

[17] Minton K. Regulatory T Cells: Subset-specific Suppression. Nat Rev Immunol (2017) 17:401. 10.1038/nri.2017.68 28627521

[18] Getnet D Grosso JF Goldberg MV Harris TJ Yen HR Bruno TC. A Role for the Transcription Factor Helios in Human CD4(+)CD25(+) Regulatory T Cells. Mol Immunol (2010) 47:1595–600. 10.1016/j.molimm.2010.02.001 PMC3060613 20226531

[19] Kim YC Bhairavabhotla R Yoon J Golding A Thornton AM Tran DQ. Oligodeoxynucleotides Stabilize Helios-expressing Foxp3+ Human T Regulatory Cells During In Vitro Expansion. Blood (2012) 119:2810–8. 10.1182/blood-2011-09-377895 PMC3327459 22294730

[20] Fu W Ergun A Lu T Hill JA Haxhinasto S Fassett MS. A Multiply Redundant Genetic Switch ‘Locks In’ the Transcriptional Signature of Regulatory T Cells. Nat Immunol (2012) 13:972–80. 10.1038/ni.2420 PMC3698954 22961053

[21] Kim HJ Barnitz RA Kreslavsky T Brown FD Moffett H Lemieux ME. Stable Inhibitory Activity of Regulatory T Cells Requires the Transcription Factor Helios. Science (2015) 350:334–9. 10.1126/science.aad0616 PMC4627635 26472910

[22] Shevach EM Thornton AM. tTregs, pTregs, and iTregs: Similarities and Differences. Immunol Rev (2014) 259:88–102. 10.1111/imr.12160 24712461 PMC3982187

[23] Thornton AM Lu J Korty PE Kim YC Martens C Sun PD. Helios+ and Helios- Treg Subpopulations are Phenotypically and Functionally Distinct and Express Dissimilar TCR Repertoires. Eur J Immunol (2019) 49:398–412. 10.1002/eji.201847935 30620397 PMC6402968

[24] Zhong H Yazdanbakhsh K. Differential Control of Helios(+/-) Treg Development by Monocyte Subsets Through Disparate Inflammatory Cytokines. Blood (2013) 121:2494–502. 10.1182/blood-2012-11-469122 PMC3612859 23365462

[25] Zhu M Xu K Chen Y Gu Y Zhang M Luo F. Identification of Novel T1d Risk Loci and Their Association With Age and Islet Function At Diagnosis in Autoantibody-Positive T1d Individuals: Based on a Two-Stage Genome-Wide Association Study. Diabetes Care (2019) 42:1414–21. 10.2337/dc18-2023 31152121

[26] Bonelli M Savitskaya A Steiner CW Rath E Smolen JS Scheinecker C. Phenotypic and Functional Analysis of CD4+ Cd25- Foxp3+ T Cells in Patients With Systemic Lupus Erythematosus. J Immunol (2009) 182:1689–95. 10.4049/jimmunol.182.3.1689 19155519

[27] Ferreira RC Simons HZ Thompson WS Rainbow DB Yang X Cutler AJ. Cells With Treg-specific FOXP3 Demethylation But Low CD25 are Prevalent in Autoimmunity. J Autoimmun (2017) 84:75–86. 10.1016/j.jaut.2017.07.009 28747257 PMC5656572

[28] Seddiki N Santner-Nanan B Martinson J Zaunders J Sasson S Landay A. Expression of Interleukin (IL)-2 and IL-7 Receptors Discriminates Between Human Regulatory and Activated T Cells. J Exp Med (2006) 203:1693–700. 10.1084/jem.20060468 PMC2118333 16818676

[29] Picca CC Larkin J3 Boesteanu A Lerman MA Rankin AL Caton AJ. Role of TCR Specificity in CD4+ CD25+ Regulatory T-cell Selection. Immunol Rev (2006) 212:74–85. 10.1111/j.0105-2896.2006.00416.x 16903907

[30] Opstelten R de Kivit S Slot MC van den Biggelaar M Iwaszkiewicz-Grześ D Gliwiński M. Gpa33: A Marker to Identify Stable Human Regulatory T Cells. J Immunol (2020) 204:3139–48. 10.4049/jimmunol.1901250 32366581

[31] Orrù V Steri M Sole G Sidore C Virdis F Dei M. Genetic Variants Regulating Immune Cell Levels in Health and Disease. Cell (2013) 155:242–56. 10.1016/j.cell.2013.08.041 PMC5541764 24074872

[32] Menning A Höpken UE Siegmund K Lipp M Hamann A Huehn J. Distinctive Role of CCR7 in Migration and Functional Activity of Naive- and Effector/Memory-Like Treg Subsets. Eur J Immunol (2007) 37:1575–83. 10.1002/eji.200737201 17474155

[33] Smigiel KS Richards E Srivastava S Thomas KR Dudda JC Klonowski KD. CCR7 Provides Localized Access to IL-2 and Defines Homeostatically Distinct Regulatory T Cell Subsets. J Exp Med (2014) 211:121–36. 10.1084/jem.20131142 PMC3892972 24378538

[34] Mailloux AW Sugimori C Komrokji RS Yang L Maciejewski JP Sekeres MA. Expansion of Effector Memory Regulatory T Cells Represents a Novel Prognostic Factor in Lower Risk Myelodysplastic Syndrome. J Immunol (2012) 189:3198–208. 10.4049/jimmunol.1200602 PMC3436939 22875800

[35] Stumpf M Zhou X Chikuma S Bluestone JA. Tyrosine 201 of the Cytoplasmic Tail of CTLA-4 Critically Affects T Regulatory Cell Suppressive Function. Eur J Immunol (2014) 44:1737–46. 10.1002/eji.201343891 PMC4051436 24648182

[36] Gordon S Taylor PR. Monocyte and Macrophage Heterogeneity. Nat Rev Immunol (2005) 5:953–64. 10.1038/nri1733 16322748

[37] Abeles RD McPhail MJ Sowter D Antoniades CG Vergis N Vijay GK. Cd14, CD16 and HLA-DR Reliably Identifies Human Monocytes and Their Subsets in the Context of Pathologically Reduced HLA-DR Expression by CD14(hi) /CD16(Neg) Monocytes: Expansion of CD14(hi) /CD16(Pos) and Contraction of CD14(lo) /CD16(Pos) Monocytes in Acute Liver Failure. Cytometry A (2012) 81:823–34. 10.1002/cyto.a.22104 22837127

[38] Du W Shen YW Lee WH Wang D Paz S Kandeel F. Foxp3+ Treg Expanded From Patients With Established Diabetes Reduce Helios Expression While Retaining Normal Function Compared to Healthy Individuals. PloS One (2013) 8:e56209. 10.1371/journal.pone.0056209 23409157 PMC3569420

[39] Golding A Hasni S Illei G Shevach EM. The Percentage of FoxP3+Helios+ Treg Cells Correlates Positively With Disease Activity in Systemic Lupus Erythematosus. Arthritis Rheumatol (2013) 65:2898–906. 10.1002/art.38119 PMC3891045 23925905

[40] Takatori H Kawashima H Matsuki A Meguro K Tanaka S Iwamoto T. Helios Enhances Treg Cell Function in Cooperation With Foxp3. Arthritis Rheumatol (2015) 67:1491–502. 10.1002/art.39091 25733061

[41] Alexander T Sattler A Templin L Kohler S Groß C Meisel A. Foxp3+ Helios+ Regulatory T Cells are Expanded in Active Systemic Lupus Erythematosus. Ann Rheum Dis (2013) 72:1549–58. 10.1136/annrheumdis-2012-202216 23264341

[42] Zóka A Barna G Somogyi A Műzes G Oláh Á Al-Aissa Z. Extension of the CD4⁺Foxp3⁺CD25(-/low) Regulatory T-cell Subpopulation in Type 1 Diabetes Mellitus. Autoimmunity (2015) 48:289–97. 10.3109/08916934.2014.992518 25523632

[43] Garg G Tyler JR Yang JH Cutler AJ Downes K Pekalski M. Type 1 Diabetes-Associated IL2RA Variation Lowers IL-2 Signaling and Contributes to Diminished CD4+CD25+ Regulatory T Cell Function. J Immunol (2012) 188:4644–53. 10.4049/jimmunol.1100272 PMC3378653 22461703

[44] Dendrou CA Wicker LS. The IL-2/CD25 Pathway Determines Susceptibility to T1D in Humans and NOD Mice. J Clin Immunol (2008) 28:685–96. 10.1007/s10875-008-9237-9 18780166

[45] Long SA Cerosaletti K Wan JY Ho JC Tatum M Wei S. An Autoimmune-Associated Variant in PTPN2 Reveals an Impairment of IL-2R Signaling in CD4(+) T Cells. Genes Immun (2011) 12:116–25. 10.1038/gene.2010.54 PMC3058680 21179116

[46] Baine I Basu S Ames R Sellers RS Macian F. Helios Induces Epigenetic Silencing of IL2 Gene Expression in Regulatory T Cells. J Immunol (2013) 190:1008–16. 10.4049/jimmunol.1200792 PMC3558938 23275607

[47] Davis AK DuBose SN Haller MJ Miller KM DiMeglio LA Bethin KE. Prevalence of Detectable C-Peptide According to Age At Diagnosis and Duration of Type 1 Diabetes. Diabetes Care (2015) 38:476–81. 10.2337/dc14-1952 25519448

[48] Zhang B Zhang X Tang FL Zhu LP Liu Y Lipsky PE. Clinical Significance of Increased CD4+CD25-Foxp3+ T Cells in Patients With New-Onset Systemic Lupus Erythematosus. Ann Rheum Dis (2008) 67:1037–40. 10.1136/ard.2007.083543 18199598

[49] Nocentini G Alunno A Petrillo MG Bistoni O Bartoloni E Caterbi S. Expansion of Regulatory GITR+CD25 Low/-CD4+ T Cells in Systemic Lupus Erythematosus Patients. Arthritis Res Ther (2014) 16:444. 10.1186/s13075-014-0444-x 25256257 PMC4209023

[50] Parackova Z Kayserova J Danova K Sismova K Dudkova E Sumnik Z. T Regulatory Lymphocytes in Type 1 Diabetes: Impaired CD25 Expression and IL-2 Induced STAT5 Phosphorylation in Pediatric Patients. Autoimmunity (2016) 49:523–31. 10.1080/08916934.2016.1217998 27560779

[51] Rosenblum MD Way SS Abbas AK. Regulatory T Cell Memory. Nat Rev Immunol (2016) 16:90–101. 10.1038/nri.2015.1 26688349 PMC5113825

[52] Mitsuiki N Schwab C Grimbacher B. What did We Learn From CTLA-4 Insufficiency on the Human Immune System? Immunol Rev (2019) 287:33–49. 10.1111/imr.12721 30565239

[53] Lindley S Dayan CM Bishop A Roep BO Peakman M Tree TI. Defective Suppressor Function in CD4(+)CD25(+) T-Cells From Patients With Type 1 Diabetes. Diabetes (2005) 54:92–9. 10.2337/diabetes.54.1.92 15616015

[54] Himmel ME MacDonald KG Garcia RV Steiner TS Levings MK. Helios+ and Helios- Cells Coexist Within the Natural FOXP3+ T Regulatory Cell Subset in Humans. J Immunol (2013) 190:2001–8. 10.4049/jimmunol.1201379 23359504

[55] Zhang R Huynh A Whitcher G Chang J Maltzman JS Turka LA. An Obligate Cell-Intrinsic Function for CD28 in Tregs. J Clin Invest (2013) 123:580–93. 10.1172/JCI65013 PMC3561819 23281398

[56] Holt MP Punkosdy GA Glass DD Shevach EM. Tcr Signaling and CD28/CTLA-4 Signaling Cooperatively Modulate T Regulatory Cell Homeostasis. J Immunol (2017) 198:1503–11. 10.4049/jimmunol.1601670 PMC5296272 28053234

[57] Irvine KM Gallego P An X Best SE Thomas G Wells C. Peripheral Blood Monocyte Gene Expression Profile Clinically Stratifies Patients With Recent-Onset Type 1 Diabetes. Diabetes (2012) 61:1281–90. 10.2337/db11-1549 PMC3331753 22403299

[58] Ren X Mou W Su C Chen X Zhang H Cao B. Increase in Peripheral Blood Intermediate Monocytes is Associated With the Development of Recent-Onset Type 1 Diabetes Mellitus in Children. Int J Biol Sci (2017) 13:209–18. 10.7150/ijbs.15659 PMC5332875 28255273

[59] Oras A Peet A Giese T Tillmann V Uibo R. A Study of 51 Subtypes of Peripheral Blood Immune Cells in Newly Diagnosed Young Type 1 Diabetes Patients. Clin Exp Immunol (2019) 198:57–70. 10.1111/cei.13332 31116879 PMC6718284

[60] McGovern JL Nguyen DX Notley CA Mauri C Isenberg DA Ehrenstein MR. Th17 Cells are Restrained by Treg Cells Via the Inhibition of Interleukin-6 in Patients With Rheumatoid Arthritis Responding to Anti-Tumor Necrosis Factor Antibody Therapy. Arthritis Rheumatol (2012) 64:3129–38. 10.1002/art.34565 22674488

[61] Kawanaka N Yamamura M Aita T Morita Y Okamoto A Kawashima M. CD14+,CD16+ Blood Monocytes and Joint Inflammation in Rheumatoid Arthritis. Arthritis Rheumatol (2002) 46:2578–86. 10.1002/art.10545 12384915

[62] Guo N Liu L Yang X Song T Li G Li L. Immunological Changes in Monocyte Subsets and Their Association With Foxp3+ Regulatory T Cells in HIV-1-Infected Individuals With Syphilis: A Brief Research Report. Front Immunol (2019) 10:714. 10.3389/fimmu.2019.00714 31024549 PMC6465566

[63] Liu L Zhang Q Chen P Guo N Song A Huang X. Foxp3+Helios+ Regulatory T Cells are Associated With Monocyte Subsets and Their PD-1 Expression During Acute HIV-1 Infection. BMC Immunol (2019) 20:38. 10.1186/s12865-019-0319-7 31651258 PMC6813100

[64] Spence A Purtha W Tam J Dong S Kim Y Ju CH. Revealing the Specificity of Regulatory T Cells in Murine Autoimmune Diabetes. Proc Natl Acad Sci U S A (2018) 115:5265–70. 10.1073/pnas.1715590115 PMC5960284 29712852

[65] Cari L Montanucci P Basta G Petrillo MG Ricci E Pescara T. Microencapsulated G3C Hybridoma Cell Graft Delays the Onset of Spontaneous Diabetes in NOD Mice by an Expansion of Gitr+ Treg Cells. Diabetes (2020) 69:965–80. 10.2337/db19-0087 32169893

