# Supplementary material for: Differences in Maturation Status and Immune Phenotypes of Circulating Helios+ and Helios− Tregs and Their Disrupted Correlations With Monocyte Subsets in Autoantibody-Positive T1D Individuals
Source: Front Immunol. 2021 May 12;12:628504. doi: 10.3389/fimmu.2021.628504 (PMC8149963; doi:10.3389/fimmu.2021.628504)
Supplement: Supplementary Figure 1 — The effect of T1D risk loci on the frequencies of Helios+ and Helios− Tregs in both healthy controls and T1D individuals, including rs2104286 in IL2RA, rs6897932 in IL-7R and rs478582 and rs1893217 in PTPN2. Con represents healthy controls. A total of 40 T1D individuals and 40 healthy controls were enrolled for the analysis. For both healthy controls and T1D individuals, comparisons between wild genotype and homozygote+ heterozygote and were performed by unpaired t test with Welch’s correction. A p value below 0.05 indicates a significant difference for a different genotype in each groups. [file Presentation_1.pptx]

## Slide 1
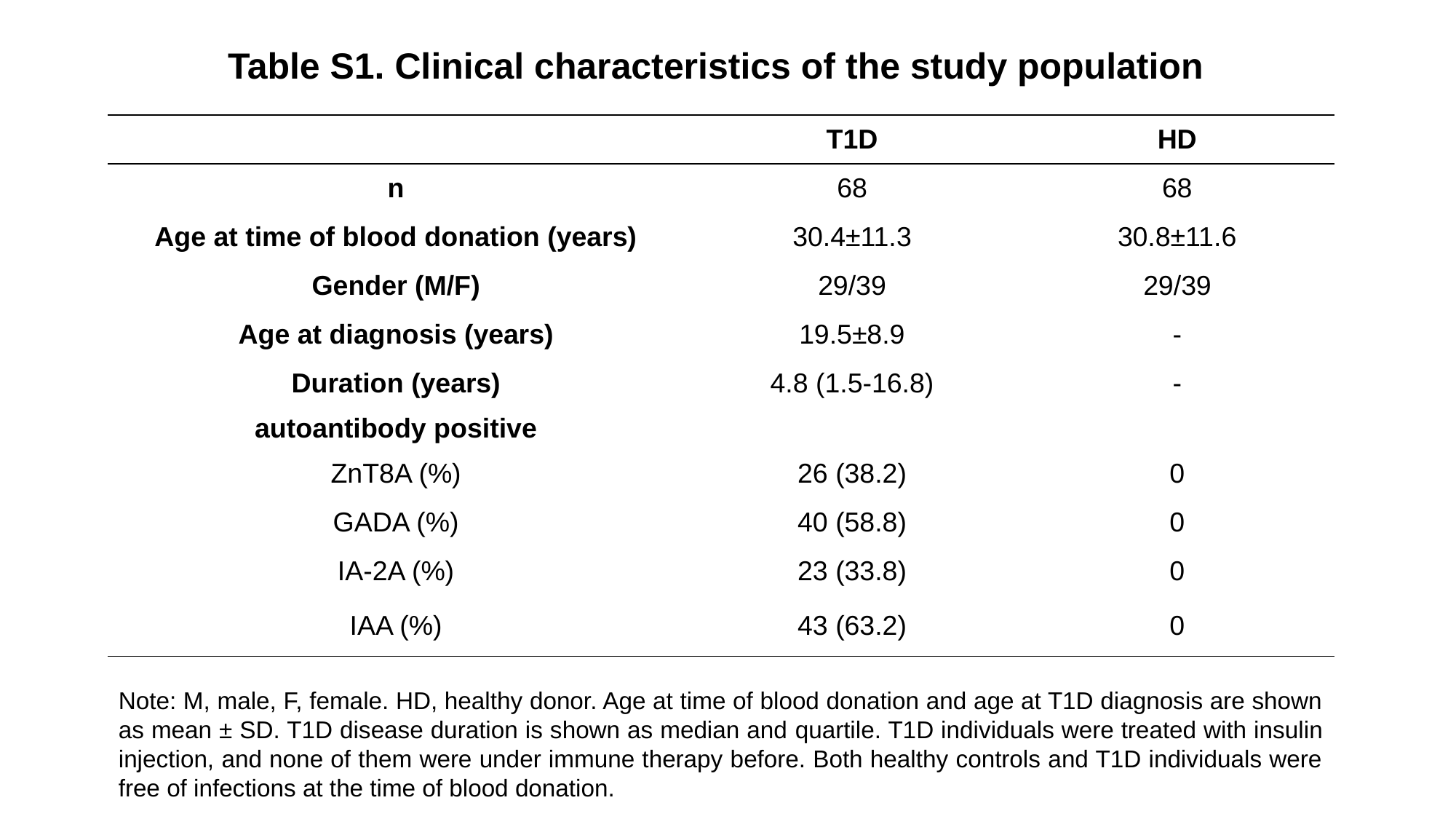

Table S1. Clinical characteristics of the study population
| | T1D | HD |
| --- | --- | --- |
| n | 68 | 68 |
| Age at time of blood donation (years) | 30.4±11.3 | 30.8±11.6 |
| Gender (M/F) | 29/39 | 29/39 |
| Age at diagnosis (years) | 19.5±8.9 | - |
| Duration (years) | 4.8 (1.5-16.8) | - |
| autoantibody positive | | |
| ZnT8A (%) | 26 (38.2) | 0 |
| GADA (%) | 40 (58.8) | 0 |
| IA-2A (%) | 23 (33.8) | 0 |
| IAA (%) | 43 (63.2) | 0 |
Note: M, male, F, female. HD, healthy donor. Age at time of blood donation and age at T1D diagnosis are shown as mean ± SD. T1D disease duration is shown as median and quartile. T1D individuals were treated with insulin injection, and none of them were under immune therapy before. Both healthy controls and T1D individuals were free of infections at the time of blood donation.

## Slide 2
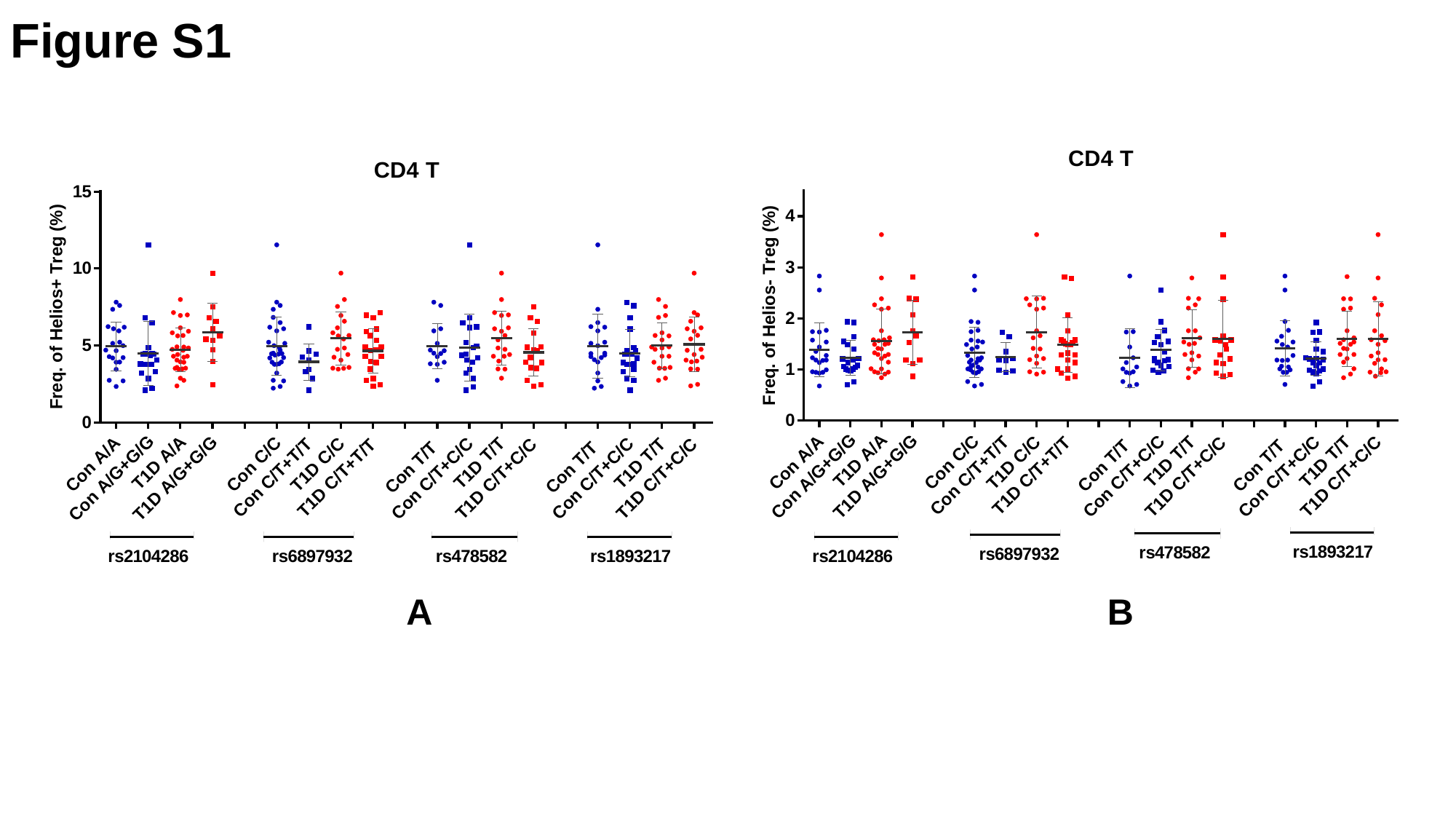

Figure S1
B
A

## Slide 3
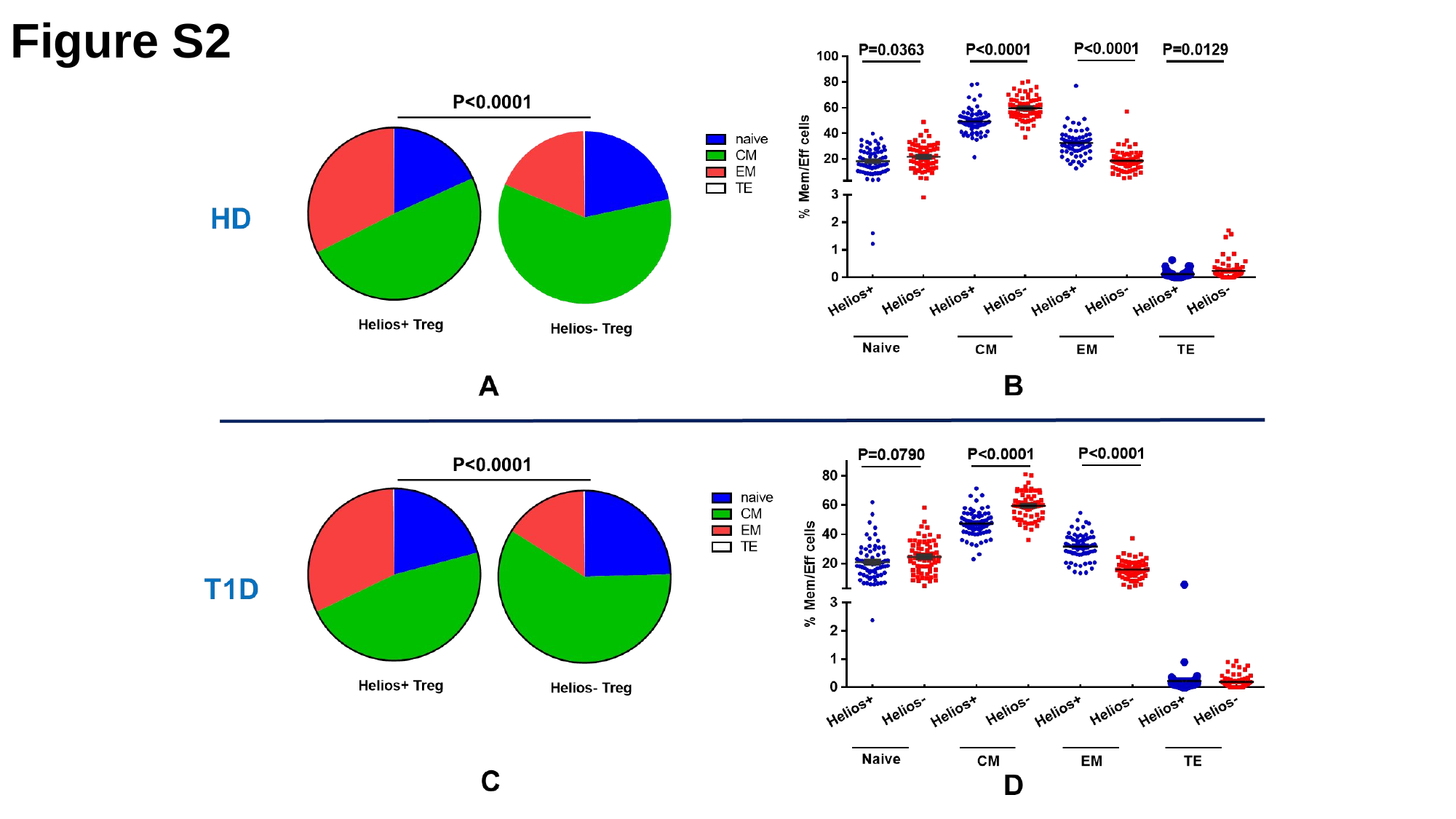

Figure S2

## Slide 4
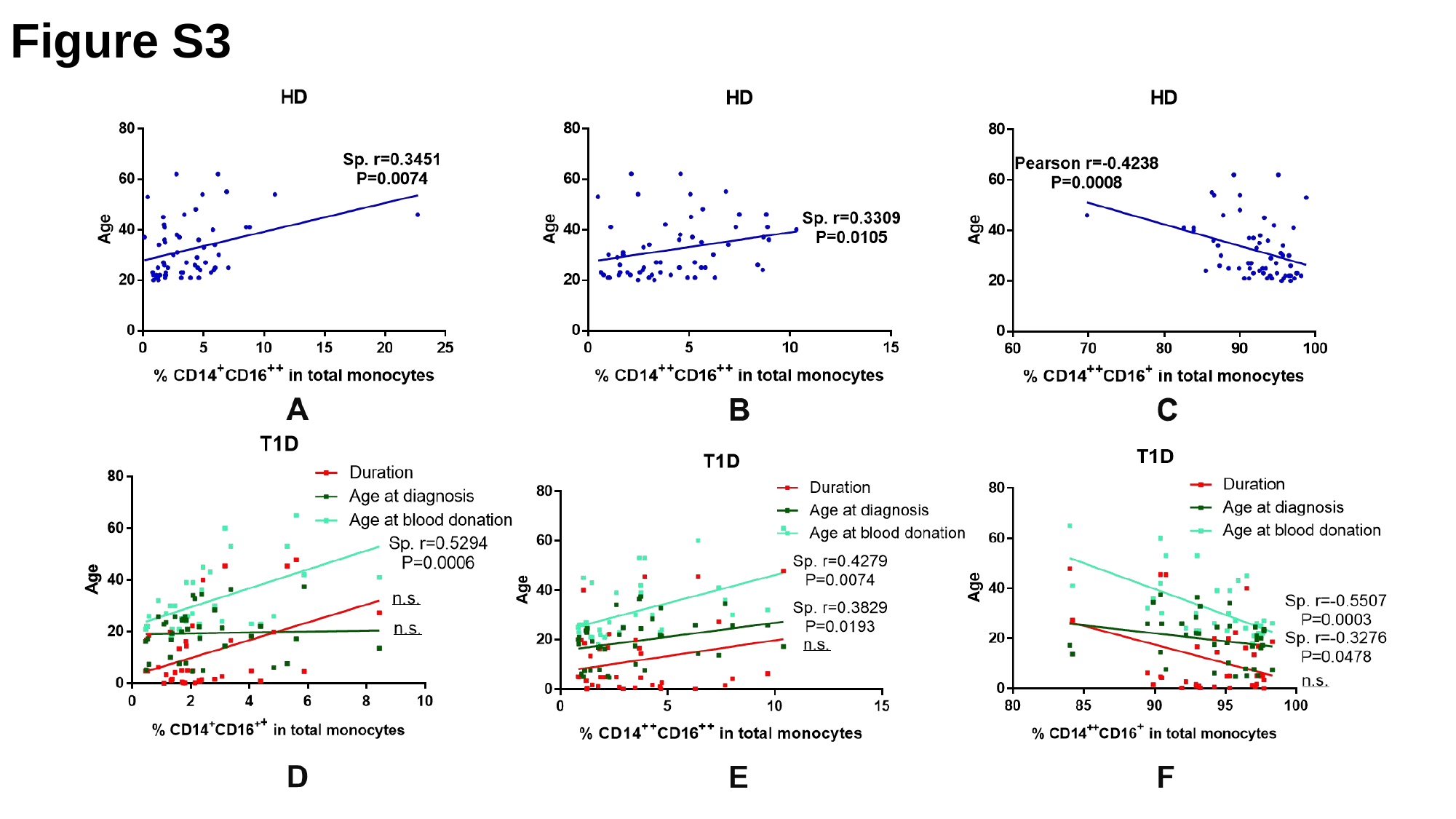

Figure S3
